# Supplementary material for: Women’s Health Information-Seeking Experiences and Preferences for Health Communications on FDA-Regulated Products: A Qualitative Study in Urban Area
Source: Int J Environ Res Public Health. 2024 Mar 9;21(3):321. doi: 10.3390/ijerph21030321 (PMC10970415; doi:10.3390/ijerph21030321)
Supplement: Supplementary file 1 [file ijerph-21-00321-s001.zip › ijerph-2833043-supplementary.pdf]

Women's Health Information-Seeking Experiences and Preferences for What and How to Receive Health Communications on FDA-Regulated Products: A Qualitative Study in Urban Area

Focus Group Guide

Focus Group Preamble

Thank you for taking part in this focus group. We are delighted that you have agreed to join us in this very important discussion today. I am [FACILITATOR NAME] and I will be starting today's discussion. But I would like for this to be a conversation among everyone. This is [NAME], who will be taking notes so we can refer to this later if we have any questions.

As [RECRUITER] may have explained to you, we are here to talk to you about your preferences for how you receive health information on some of the products the US Food and Drug Administration (also known as the FDA) regulates. We use the term health information to refer to messages that increase awareness of a health issue, including food and drug safety information. Today, we want to discuss how you would prefer the FDA to communicate health information to you.

There are two parts to our discussion today. During the first part, we want to learn from you how you go about finding health information, and what you do with this information. During the second half of our discussion, we want to get your opinions about different types of FDA health messages. Your opinions and feedback are valuable to the FDA because they are committed to making sure that you have access to information that is clear and understandable, and that it is information that you can use when making decisions about your health. It will also improve their communication by basing it on women's preferences. For example, are FDA communications easy to find? Easy to read? Do they give you the information you're looking for?

Your feedback will help FDA respond effectively to the health information needs of women just like you. Your opinions also will help the FDA improve how they communicate health information to you that you will find useful. We will not discuss *your personal health issues or treatments*. The focus today is to learn how you go about finding health information.

Before we continue, I want to stop and see if you have any questions about what I have said so far.
Answer any questions.

Before we begin our discussion, I would first like to go over a few "housekeeping" guidelines.

In a focus group it is really important that you express yourself openly. There is no right or wrong answer. We want to know what you think. I am here to guide you, but you should feel free to express your opinion and discuss it among yourselves. If you would like to add to an idea, or if you have an idea that is different from someone else, feel free to jump in. You do not need to wait for me to call on you to talk, but of course only one person should speak at a time.

I want to remind you that your participation in this discussion is *voluntary* and you can choose *not* to respond to any question for any reason.

Also, we ask you to keep what will be discussed today within this group and not to share the discussion with others who did not participate. This is to make everyone feel comfortable sharing their honest opinions with the group.

At this time, I will allow a few moments for you to read the participant information sheet that describes this study and what you will be asked to do if you agree to join this study.
Give participants 10 minutes to review the sheet. Answer any questions that may arise. Discuss 1-1 at the individual's request. Ask if there are any questions or things that need clarification.

Do you give verbal consent to participate in this focus group?

Confirm that participants provided verbal consent. Moderator signs and dates the consent log form as documentation that all participants have agreed to join the study.

We would like to record this discussion today as a back-up to make sure our notes are correct. What you say will *not* be linked to your name in any report and the recording will be destroyed after we have analyzed the discussions. If at any time you do not want your comment to be recorded, please let [NAME] know and we will turn off the recorders while you are speaking.

Do I have your permission to record our discussion? *Obtain a verbal agreement. If anyone does not want to be recorded – the focus group discussion will not be recorded.*

Please silence your cell phones and other electronic devices. You might also notice that I may repeat or ask questions about some of the comments that you make throughout the discussion today. I will do this not only to ensure that we capture everything that is said in the group, but also to confirm that I understand what you have said.

Are there any questions before we get started? *Answer any questions.*

Introduction

Let's first go around the room and have each of you state your first name. If you do not wish to reveal your name, you can use an alias. When you introduce yourself, tell us what the last piece of health-related information was you heard or obtained and where you got this information.

Health information seeking intentions and behaviors with respect to FDA-regulated products

For the first half of our discussion, I have a few questions about what you do when you want to find more information about either your own health or the answer to a health question in general. Health is a large topic, so for today's discussion, let's focus on 4 topics: food, drugs, vaccines and medical devices, such as an insulin pump or a pacemaker.

Perhaps you have had a question about health information on the front of a food package, a prescription medication, a flu vaccine, or a medical device.

If participants start talking about their health conditions, treatments, insurance, etc, steer the conversation back to the process of finding health information.

1. When you had this health concern, did you try to find information to answer your questions? You don't need to talk specifically about any health issues, but just whether you tried to find information about this.

   Probe:
   - If no, why not (e.g., fears, time, not sure where to find information)
   - If yes, why (e.g., motivations; health-related goals; discussion with provider, family, friends)
   - Would your responses differ depending on if it is for Drugs? Vaccines? Medical devices? Health information on the front of a food package?
   - Is there a health topic that you feel you do not need information for?

2. If you did look up information to answer your question, where you did look for this information?

   Probe:
   - Sources of information: family, friends; physicians, pharmacists, other healthcare providers; pharmaceutical industries; patient support groups; medical societies; ministers, communities, senior centers; product website, the internet, social media; journal articles; news.
   - Would your responses differ depending on if it is for Drugs? Vaccines? Medical devices? Health information on the front of a food package?
   - Are these sources of information the ones that you rely on the most? If yes, why are they your trusted sources of information? If not, what are your trusted sources of information?
   - How frequently, if at all, do you seek information from the FDA website? When you visit the FDA website, if at all, do you find the information useful and easy to access? If not, why not?

3. Thinking about the types of information that you did find, was this information useful (e.g., testimonials, data, type of messenger (peer, provider, etc.)?

   Probe:
   - If yes, what made this information useful to you?
   - If not, what would you need to make it more helpful?

- Would your responses differ depending on if it is for Drugs? Vaccines? Medical devices? Health information on the front of a food package?

Let's now consider a scenario where you have gathered your health information, and you now want to make a decision based on this information. For example, this decision could be to evaluate the types of food you purchase and eat, whether to begin a particular medication treatment, whether to get the flu vaccine, or what type of insulin pump to use.

4. Assuming that you've learned about the different options that you can take to improve your health, what source of information is most helpful or influential to you in making a health-related decision? (e.g., family, friends, physicians, pharmacists, other healthcare providers, pharmaceutical industries, patient support groups, medical societies, ministers, communities, senior centers, FDA website, product website, the internet, social media, journal articles, news)
   Probe:
   - What source(s) do you find most helpful?
   - Would your responses differ depending on if the information is for Drugs? Vaccines? Medical devices? Health information on the front of a food package?

5. Have you ever tried to find answers to a health question, but couldn't find enough information about the topic?
   Probe:
   - If yes, what was the reason for this? (e.g., information lacking or needed improvement, overwhelmed with too much information, don't know where and how to find this information)
   - Did you decide to make a decision about your health even though you could not find enough information about the topic? If so, why or why not? If yes, how?

Older women's perceptions about FDA's health communication regarding FDA-regulated products

Now, for the second part of our discussion, I want to show some examples of how health information can be shared with women like you. These represent different ways to communicate health information to the public, such as [add in examples of types of communication].

After I show you each example of a health communication tool, I'll ask you a few questions to get your opinions about each type of communication. I won't ask you questions about the words, graphics, images, or colors. Instead, I'm very interested in hearing what you think about receiving health information through each type of communication.

Note, if participants speak about content, message, or design, redirect discussion to type of communication as a way to receive health information.

Let’s take a look at the first [type of communication]. This is an example of [type of communication, add in brief description].

6. What are your thoughts about receiving health information in this [type of communication] format?
   Probe:
   - Does this [type of communication] appeal to you as a way to receive health information? If so, what makes it appealing? If not, what are the reasons (e.g., can’t read well, need help to understand)?
   - What would make [type of communication] more helpful when you are looking for health information (e.g., time, access to technology, balanced information on both benefits and risks, large font size, short, comprehensive, patient experience data, up to date)?
   - Would [type of communication] be the most informative way to receive information about Drugs? Vaccines? Medical devices? Health information on the front of a food package?

7. Thinking about [type of communication] as a way to receive health information, would you recommend [type of communication] to others?
   Probe:
   - Whom would you recommend this to (e.g., age, gender, cultural groups)?
   - What are some reasons why you would recommend this type of communication to others? Reasons for why you would not recommend it to others?

Repeat questions #6 and #7 for each type of communication.

8. Do you have a preference for one type of communication format over the others when you think of…
   a. Drugs?
   b. Vaccines?
   c. Medical Devices?
   d. Health information on the front of the food package?

9. Is there any other type of communication that you would prefer to receive but we have not discussed today?

CLOSURE

Those are all the questions we had for you. Before we end, thinking back to our discussion, is there anything that comes to mind that we didn’t already talk about?

Thank you for participating. You have all worked hard today and we have learned a great deal from you.

We appreciate your help with this important topic.

If any additional thoughts come up that you would like to convey, please feel free to contact the [PI NAME} by email or phone, as indicated on your information sheet [PI email, phone].
